# Effect of Chronic Kidney Disease on Changes in Vasopressin System Expression in the Kidney Cortex in Rats with Nephrectomy

**DOI:** 10.1155/2018/2607928

**Published:** 2018-06-14

**Authors:** Katarzyna Czarzasta, Agnieszka Cudnoch-Jedrzejewska, Longin Niemczyk, Robert Wrzesien, Marzanna Tkaczyk, Liana Puchalska, Marek Saracyn, Wawrzyniec Zmudzki, Stanisław Niemczyk

**Affiliations:** ^1^Department of Experimental and Clinical Physiology, Laboratory of Centre for Preclinical Research, Medical University of Warsaw, Warsaw, Poland; ^2^Department of Nephrology, Dialysis and Internal Medicine, Medical University of Warsaw, Warsaw, Poland; ^3^Central Laboratory of Experimental Animals, Laboratory of Centre for Preclinical Research, Medical University of Warsaw, Warsaw, Poland; ^4^Department of Endocrinology and Isotope Therapy, Military Institute of Medicine, Warsaw, Poland; ^5^Department of Internal Medicine, Nephrology and Dialysis, Military Institute of Medicine, Warsaw, Poland

## Abstract

It is believed that the vasopressinergic system plays an important role in the pathogenesis of chronic kidney disease (CKD). The aim of this study was to evaluate the effect of CKD on changes in vasopressin system expression in the kidney cortex in rats with nephrectomy. The study was performed on 4 groups of Sprague Dawley (SPRD) rats: a control group (*CN*), 1/2 nephrectomy (*N1/2*), 2/3 nephrectomy (*N2/3*), and 5/6 nephrectomy (*N5/6*). Blood and the kidney cortex were collected to evaluate plasma copeptin concentrations and mRNA expressions of V1a vasopressin receptors (V1aR) and V2 vasopressin receptors (V2R) and V1aR, V2R, and aquaporin 2 (AQP2) protein levels. V1aR and V2R mRNA expression in the kidney cortex was significantly lower in the* CN* group compared with the other groups. In contrast, the V1aR, V2R, and AQP2 protein levels were significantly higher in the* CN* group compared with all of the nephrectomized groups. Plasma copeptin concentration was significantly lower in the* CN* group than in the nephrectomized groups. CKD caused significant changes in the expression of the vasopressinergic system. Further research is needed to explain the mechanisms of the impact of the vasopressinergic system on the kidney in CKD.

## 1. Introduction

Chronic kidney disease (CKD) is a common, progressive disease, which leads to irreversible loss of function of this organ in all age groups [1]. The prevalence of CKD is not precisely known. It is estimated that it is approximately 7% in young adults and 35% in the elderly [2].

The pathogenesis of chronic kidney disease is complicated and the disease develops over many years, often with a long latent period [3]. It leads to rapidly progressive renal failure and cardiovascular system diseases, including heart failure [4].

It has long been known that centrally released vasopressin (AVP) regulates urine concentration, reabsorption of water in the renal tubules, and, to a lesser extent, reabsorption of sodium [5–8]. Vasopressin acts through the V1a (V1aR), V1b (V3; V1bR), and V2 (V2R) receptors. V1a receptors are present in mesangial cells, efferent arterioles, and renal tubules [9]. The V1b receptors are located only in the core of the kidney, and their function has not yet been established [10].

In the kidney, the most common vasopressin receptor is the V2 receptor [11]. V2R has been detected, for example, in macula densa cells, the thick ascending loop of Henle, the distal tubules, and collecting ducts [12].

It has been shown that increased activity of the vasopressinergic system may have harmful effects on the kidneys, causing increased blood pressure, increased glomerular filtration rate, increased renin release, and mesangial cells expansion [13]. Both in experimental and in clinical studies, it has been shown that the V2R are involved in the process of albuminuria [14].

In recent years, attention was drawn to copeptin, which derived from provasopressin—the precursor of vasopressin. Similarly to AVP, the copeptin concentration in plasma fluctuates due to changes in plasma osmolarity [15]. Copeptin, in contrast to vasopressin, is a substance more stable in plasma and its concentration does not vary according to age [16]. The relationship between microalbuminuria and copeptin concentration in the plasma of healthy subjects is described in the population study PREVEND [17]. A significant association between the reduction of copeptin plasma concentration and the deterioration of renal function has been shown [13, 18]. It was also found that there is a significant increase in copeptin plasma concentration in patients with chronic heart failure, hypertension, and/or type 2 diabetes whose renal function was preserved [19–21].

Based on data from the literature, it is known that in patients with CKD there is a loss of glomeruli in the kidney cortex [22].

Therefore,** the aim of this study** was to evaluate the effect of chronic kidney disease on changes in vasopressin system expression (mRNA and protein levels of V1a and V2 receptors and the protein level of aquaporin 2 (AQP2)) in the kidney cortex and in addition to assess the usefulness of plasma copeptin concentrations in the course of chronic kidney disease in rats with nephrectomy.

## 2. Materials and Methods

**Figure 1:**
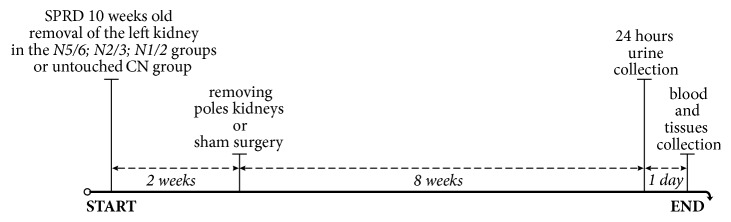
Design of the study. SPRD: Sprague Dawley rats.

Tests were carried out on 28 ten-week-old Sprague Dawley rats (SPRD/Mol/Lod), obtained from Central Laboratory of Experimental Animals, Medical University of Warsaw. The rats were housed in individual cages under monitored conditions (temperature 22–25°C; humidity 40–60%; 12-hour light-dark cycle), and food and water were available* ad libitum*. All experimental procedures were approved by The II Local Ethics Committee in Warsaw at Faculty of Biology University of Warsaw (19/2013), and they were conducted at the Department of Experimental and Clinical Physiology, Laboratory of Centre for Preclinical Research, Medical University of Warsaw. The rats were divided into 4 groups: control group (*CN*; n=7), 1/2 nephrectomy group (*N1/2*; n=7), 2/3 nephrectomy group (*N2/3*; n=6), and 5/6 nephrectomy group (*N5/6*; n=8), which were subjected to the following procedures (Figure 1).

### 2.1. Surgical Procedures

#### 2.1.1. The 5/6 Nephrectomy (N5/6)

The 5/6 nephrectomy was performed on ten-week-old rats under general anesthesia (Ketamine 10 mg/100 g body weight (b. wt.) (Vetoquinol), intraperitoneal injection (i.p.); Xylazine 1 mg/100 g b. wt., i.p.) (Vetoquinol). An incision in the skin in the lumbar area on the left, parallel to the spine, was made after the immobilization of the animal in the prone position. The left kidney was removed in its entirety after ligation of vessels and ureter. Two weeks after the first intervention, a second operation was performed. In the prone position, the skin was cut in the lumbar region on the right, parallel to the spine, and the right kidney was recovered and, after ligation, the top and bottom poles (superior and interior segments) were removed to cause kidney damage. The wound was closed by surgical suture (Vicryl 4.0, Ethicon).

#### 2.1.2. The 2/3 Nephrectomy (N2/3)

The 2/3 nephrectomy was proceeded similarly to the 5/6 nephrectomy, but leaving the upper pole (superior segment) of the right kidney intact.

#### 2.1.3. The 1/2 Nephrectomy (N1/2)

In animals treated with 1/2 nephrectomy, the left kidney was removed, and the right was gently touched by a needle. The wound was closed by surgical suture (Vicryl 4.0, Ethicon).

#### 2.1.4. Control Group (CN)

The rats were not subjected to any surgical procedure.

At the end of each surgical procedure, the animals were given an analgesic (Buprenorphine chloride 3 g/100 g b. wt., i.p.; 5.95 nmol/ml, twice daily for 2–3 days) and an antibiotic (Penicillin, Polfa 10,000 IU/100 g b. wt., i.p.; 0.047 mmol/ml).

### 2.2. 24-Hour Urine Collection

24-hour urine collection was performed two months after the last surgery to confirm chronic kidney disease. In order to collect urine samples rats were placed for 24 hours in a metabolic cage. Then urine samples were placed in sterile tubes and transported to the Animals Diagnostic Laboratory (LAB-WET) for further researches. Urine samples were transported in 4°C.

### 2.3. Blood Sampling and Tissue Harvesting

Two months after the last surgery, 4 ml of blood from the right ventricle, under general anesthesia (Ketamine 10 mg/100 g b. wt., i.p.; Xylazine 1 mg/100 g b. wt., i.p.), through the second or third intercostal space, was collected for biochemical tests.

After collecting the blood, the animals were euthanized by intraperitoneal injection of a lethal dose of ketamine in order to collect the kidneys cortex. The fragments of kidneys cortex were frozen in liquid nitrogen and then stored in a deep freezer (−80°C) until analysis.

### 2.4. Biochemical Urine Tests

The urine collected from the rats was examined; the concentrations of urea, sodium, creatinine, and protein were checked in collaboration with the LAB-WET. In addition, the osmolarity of the urine was rated using an 800 CLG Osmometer (Trident Med).

### 2.5. Biochemical Blood Tests

The plasma concentrations of creatinine, urea, and sodium were evaluated in collaboration with the Animals Diagnostic Laboratory (LAB-WET). Plasma osmolarity was rated using an 800 CLG Osmometer (Trident Med). Estimated glomerular filtration rate (eGFR) was calculated with the following formula [23]:

(1) eGFR=creatinine  urine  concentration mg/100mlcreatinine  plasma  concentration mg/100ml×urine  volume ml/min

Copeptin, cystatin C (a marker of chronic renal failure), and N-terminal brain natriuretic peptide (NT-proBNP) (a marker of heart failure) plasma concentrations were checked using enzyme-linked immunoassays (ELISA) (Rat Vasopressin-neurophysin 2-copeptin ELISA Kit, Wuhan ElAab Science Co.; Mouse/Rat Cystatin C Immunoassay, R&D Systems, Inc.; Rat NT-proBNP ELISA Kit, Wuhan ElAab Science Co.).

### 2.6. mRNA Expression of V1a and V2 Receptors (Real-Time PCR)

The fragments of kidneys cortex were homogenized in TRIzol® Reagent (Ambion, Life Technologies) using a homogenizer TissuLyser LT (Qiagen). Subsequently, mRNA was extracted using the PureLink RNA Mini Kit (Ambion, Life Technologies). Then, multiplex reactions were carried out. The reaction mixture contained TaqMan RNA-to-CT 1-Step Kit, primers for the gene of interest (rat V1a receptor: Applied Biosystems gene symbol* Avpr1a*, accession number Rn00583910_m1; forward 5′- GCCTCAGGACCAGACAGAAG - 3′ reverse 5′ – AATCACTGCCAGCACAGC - 3′; rat V2 receptor: Applied Biosystems gene symbol* Avpr2*, accession number Rn00569508_g1; forward 5′ – ATGCCTCCTCCTACATGATCC - 3′ reverse 5′ – AGGGCAATCCAGGTGACATAG - 3′) labelled with FAM reporter dye, primers for GAPDH (rat GAPDH; Applied Biosystems gene symbol* Gapdh*, accession number Rn01775763_g1; forward 5′ – AATGGTGAAGGTCGGTGTGAAC - 3′ reverse 5′ – AGGTCAATGAAGGGGTCGTTG - 3′) labelled with VIC, RNA, and RNase-free water (Life Technologies). The final volume of reaction mixture (50 *μ*l) was subjected to proliferation under conditions: 15 seconds at 95°C and 1 minute at 60°C for 40 cycles in a ViiA™ 7 Real-Time PCR System thermocycler (Applied Biosystems). The relative gene expression was given, on the basis of estimations of the values of the delta cycle threshold (ΔCt), as relative amounts to the endogenous control.

### 2.7. Protein Level of V1a and V2 Receptors and Aquaporin 2 (AQP2) (Western Bot)

The kidney cortex was homogenized in RIPA lysis buffer which contained 10 mM Tris-HCl, pH 7.4; 100 mM NaCl; 1 mM EDTA; 1 mM EGTA; 1% Trion X-100; 10% glycerol; 0.1% SDS; 1mM PMSF and peptidase inhibitors leupeptin and aprotinin (Halt™ Protease and Phosphatase Inhibitor Single-Use Coctail, EDTA-Free, Thermo Fisher). Then homogenates were centrifuged and proteins were determined in a supernatant by the Bradford method (Sigma Aldrich) using BSA (bovine serum albumin; Sigma Aldrich) as a standard. Probes containing 10 *μ*g/*μ*l of total protein were separated on 8% SDS-polyacrylamide gels. Renal cortex homogenates from each rat with a given experimental group were independently scored for each SDS-polyacrylamide gel. For each of the tested proteins (V1aR, V2R, AQP2, and GAPDH), each of the samples was applied in duplicate to two independent SDS-polyacrylamide gels. Separated proteins were transferred into PVDF membranes (Trans-Blot® Turbo™ RTA Mini PVDF Transfer Kit; Bio-Rad) by using Trans-Blot® Turbo™ Transfer System (Bio-Rad). The PVDF membranes were incubated for 1 h with primary rabbit polyclonal antibody against V1aR (sc-30025; Santa Cruz Biotechnology), primary rabbit polyclonal antibody against V2R (ab109326; Abcam), primary mouse monoclonal antibody against AQP2 (sc-515770; Santa Cruz Biotechnology), and secondary antibody: goat anti-rabbit conjugated to Horseradish Peroxidase (HRP) (sc-2004; Santa Cruz Biotechnology) and mouse IgG kappa binding protein m-IgG*κ* BP conjugated HRP (sc-516102; Santa Cruz Biotechnology). For loading control, the blots were stripped and reprobed for mouse monoclonal GAPDH antibody (sc-47724; Santa Cruz Biotechnology) and mouse IgG kappa binding protein (m-IgG*κ* BP) conjugated to Horseradish Peroxidase (HRP) (sc-516102; Santa Cruz Biotechnology). The specific bands were visualized with colorimetric directly on the PVDF membrane by means placed in Amplified Opti-4CN Substrate Kit (Bio-Rad). Band intensity was quantified by the ChemiDoc Imaging Systems (ChemiDoc™ MP System, Bio-Rad). V1aR, V2R, and AQP2 protein expression was normalized with GAPDH to control for the amount of protein loading and transfer and expressed as a relative ratio. In order to normalize the results, the same GAPDH result was used for each of the tested proteins. The levels of each of the tested proteins, including GAPDH, were presented as the mean of all homogenized fragments of the kidney cortex taken from each rat in the individual experimental groups (*CN*, n = 7;* N1/2*, n = 7;* N2/3*, n = 6;* N5/6*, n = 8) in two independent replicates.

### 2.8. Statistical Analysis

Statistical analysis was performed using Statistica 12 software. Comparisons of average values of individual indicators were made using parametric tests (ANOVA single and multivariate normal distributions) and nonparametric equivalents of these tests for distributions other than normal (ANOVA Kruskal-Wallis multiple comparison average ranks and Mann–Whitney U test for independent samples and Friedman ANOVA for dependent samples). The distribution was checked for normality using the Shapiro-Wilk W test and homogeneity of variance using Levene's test (ANOVA). Tables and graphs show average values of analyzed parameters and their standard errors (± SE). Differences medium was considered statistically significant for P<0.05.

## 3. Results

### 3.1. Characteristics of Animals

The weight of rats did not differ significantly (*CN*: 380 ± 9g;* N1/2*: 404 ± 14g*; N2/3*: 391 ± 15g*; N5/6*: 426 ± 8g).

### 3.2. Biochemical Parameters in the Urine

Table 1 shows the results of urine. The volume of urine collected over 24 hours was significantly higher in the* N5/6* group in comparison with the other groups of rats (*N5/6 versus CN*, P<0.001;* N5/6 versus N1/2*, P<0.01;* N5/6 versus N2/3*, P<0.05). The osmolarity of urine was significantly lower in the* N5/6* group compared with the other groups of rats (*N5/6 versus CN*, P<0.01;* N5/6 versus N1/2*, P<0.001;* N5/6 versus N2/3*, P<0.01). There was significantly higher urine osmolarity in the* N1/2* group in comparison with the* N2/3* group (P<0.05). The urine concentration of urea was significantly lower in the* N5/6* group compared with another group of rats (P<0.001) and in the* N2/3* compared with the* CN* group (P<0.001) and in the* N1/2* group in comparison with the* CN* group of rats (P<0.01). The observed concentration of sodium in the urine was significantly lower in the* N5/6* group in comparison with the* N1/2* group (P<0.01) and in comparison with the* CN* group of rats. The urine concentration of creatinine was significantly lower in the* N5/6* group compared with another group of rats (P<0.001). The urine concentration of protein was significantly higher in the* N5/6* group compared with the* N1/2* group (P<0.05) and in comparison with* CN* group (P<0.001). The protein to creatinine ratio in urine was significantly higher in the* N5/6* group in comparison with the* N1/2* group (P<0.05) and in comparison with the* CN* group (P<0.001). The protein to creatinine ratio was also significantly higher in the* N2/3* group in comparison with the* CN* (P<0.05).

### 3.3. Biochemical Parameters in the Plasma

**Figure 2:**
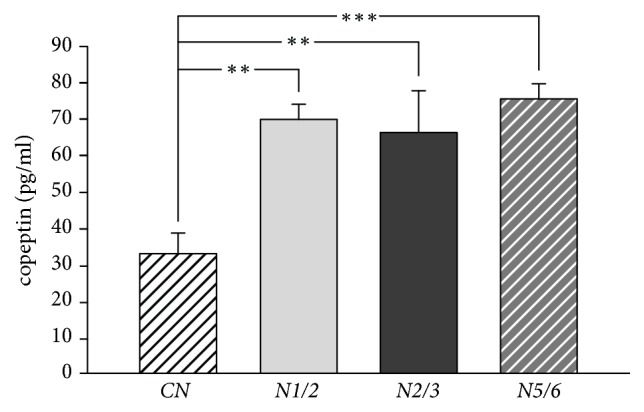
Copeptin concentration in plasma in rats with 5/6 nephrectomy, 2/3 nephrectomy, 1/2 nephrectomy, or control group.* CN*: control group;* N1/2*: 1/2 nephrectomy group,* N2/3*: 2/3 nephrectomy group;* N5/6*: 5/6 nephrectomy group. Means ± SE are shown. *∗∗*P<0.01; *∗∗∗*P<0.001.

Table 2 shows the results examination of biochemical parameters in the plasma. The osmolarity of the plasma was significantly lower in the* CN* group in comparison with the other group of rats (*CN versus N5/6*, P<0.05;* CN versus N2/3*, P<0.01;* CN versus N1/2*, P<0.05). The plasma urea concentration was significantly higher in the* N5/6* group in comparison with the* N1/2* group (P<0.05) and in comparison with the* N2/3* group (P<0.001). These parameters were also significantly higher in the* N2/3* group in comparison with the* N1/2* group (P<0.01). The plasma concentrations of sodium ions were significantly higher in the* CN* group in comparison with the* N1/2* group (P<0.05). Plasma creatinine concentration was significantly higher in the* N5/6* group compared with the* N2/3* group (P<0.001) and compared with the* N1/2* group (P<0.01). The values of estimated glomerular filtration rate (eGFR) were significantly lower in the* N5/6* group compared with the other group (*N5/6 versus N2/3*, P<0.01;* N5/6 versus N1/2*, P<0.001;* N5/6 versus CN*, P<0.001). These parameters were also significantly higher in* CN* group in comparison with the* N2/3* group (P<0.05) and in comparison with the* N1/2* group (P<0.05). Plasma concentration of cystatin C was significantly higher in the* N5/6* group in comparison with the* CN *group (P<0.001) and with the* N1/2* group (P<0.01) and with the* N2/3* group (P<0.01). Significantly higher plasma levels of cystatin C in the* N2/3* group compared with the* CN *group were found (P<0.05). Plasma concentrations of NT-proBNP were significantly higher in* N5/6* group in comparison with the* CN* group (P<0.05). There were no significant differences in the plasma concentrations of NT-proBNP between the other groups of animals. Plasma concentration of copeptin was significantly higher in the* N5/6* group in comparison with the* CN* group (P<0.001). Plasma concentration of copeptin was also significantly higher in the* N2/3* group and in the* N1/2* group in comparison with the* CN* group (P<0.01; P<0.001, respectively) (Figure 2).

### 3.4. mRNA Expression of V1a and V2 Vasopressin Receptors

**Figure 3:**
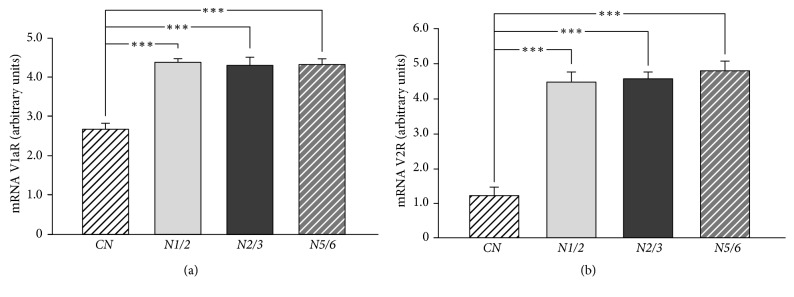
mRNA expression of V1a and V2 vasopressin receptors. (a) V1a vasopressin receptor mRNA expression in the kidney cortex in rats with 5/6 nephrectomy, 2/3 nephrectomy, 1/2 nephrectomy, or control group.* CN*: control group;* N1/2*: 1/2 nephrectomy group,* N2/3*: 2/3 nephrectomy group;* N5/6*: 5/6 nephrectomy group; V1aR: V1a vasopressin receptor. Means ± SE are shown. *∗∗∗*P<0.001. (b) V2 vasopressin receptor mRNA expression in the kidney cortex in rats with 5/6 nephrectomy, 2/3 nephrectomy, 1/2 nephrectomy, or control group. V2R: V2 vasopressin receptor. Other abbreviations as in (a). Means ± SE are shown. *∗∗∗*P<0.001.

It has been shown that mRNA expression of vasopressin mRNA V1aR and V2R in the kidney cortex was significantly lower in the* CN* group in comparison with the other groups of rats (P<0.001) (Figures 3(a) and 3(b)).

### 3.5. Protein Level of V1a and V2 Vasopressin Receptors and AQP2

**Figure 4:**
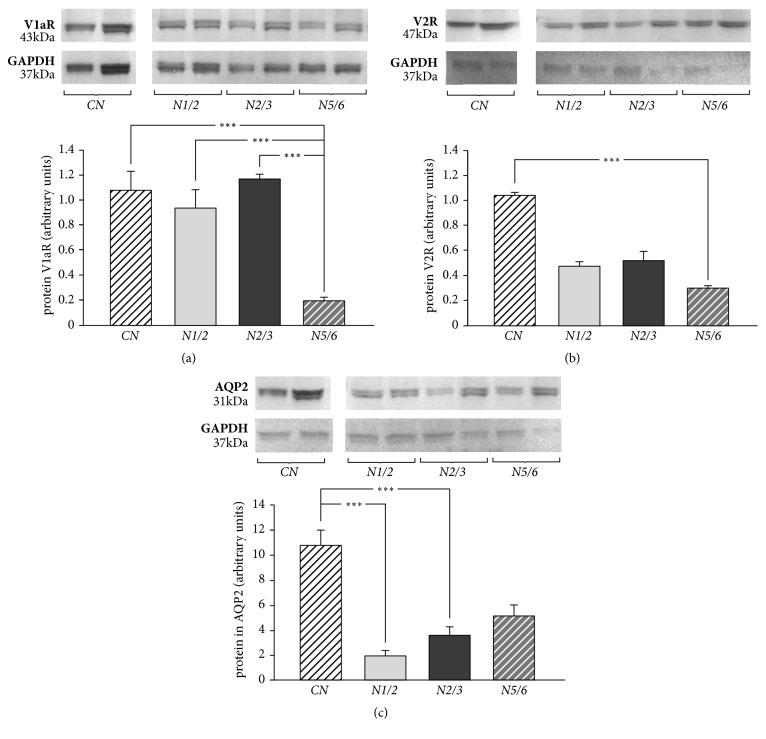
Protein level of V1a and V2 vasopressin receptors and aquaporin 2. (a) V1a vasopressin receptor protein level in the kidney cortex in rats with 5/6 nephrectomy, 2/3 nephrectomy, 1/2 nephrectomy, or control group.* CN*: control group;* N1/2*: 1/2 nephrectomy group,* N2/3*: 2/3 nephrectomy group;* N5/6*: 5/6 nephrectomy group; V1aR: V1a vasopressin receptor. The bands represent two independent samples from each group of rats. Means ± SE are shown. *∗∗∗*P<0.001. (b) V2 vasopressin receptor protein level in the kidney cortex in rats with 5/6 nephrectomy, 2/3 nephrectomy, 1/2 nephrectomy, or control group. V2R: V2 vasopressin receptor. Other abbreviations as in (a). The bands represent two independent samples from each group of rats. Means ± SE are shown; *∗∗∗*P<0.001. (c) Aquaporin 2 protein level in the kidney cortex in rats with 5/6 nephrectomy, 2/3 nephrectomy, 1/2 nephrectomy, or control group. AQP2: aquaporin 2. Other abbreviations as in (a). The bands represent two independent samples from each group of rats. Means ± SE are shown; *∗∗∗*P<0.001.

It has been shown that protein level of vasopressin V1aR in the kidney cortex was significantly lower in the* N5/6* group in comparison with the other group of rats (P<0.001) (Figure 4(a)). The protein level of vasopressin V2R in the kidney cortex was significantly lower in the* N5/6* group in comparison with the* CN* group (P<0.001) (Figure 4(b)). The AQP 2 protein level was significantly lower in the* N2/3* group than in the* CN* group (P<0.001). This parameter was also significantly lower in the* N1/2* group compared to the* CN* group (P<0.001) (Figure 4(c)).

## 4. Discussion

There is still little data published regarding the expression and role of the vasopressinergic system in the pathogenesis and progression of chronic kidney disease. The assessment of copeptin plasma concentrations as a diagnostic and prognostic marker in the animal model of CKD was also not a subject of previous studies. The results presented in this study were derived from rats with 5/6 nephrectomy. The development of chronic kidney disease in these animals was confirmed by biochemical urine and blood tests. eGFR values were significantly lower and plasma cystatin C concentration was significantly higher in rats with 5/6 nephrectomy than the other groups of rats.

Recently, special attention is being given to copeptin as a potential marker for chronic kidney disease. In the present study, plasma copeptin concentration was significantly lower in the* CN* group than in the other groups of animals studied. There were no significant differences in plasma copeptin concentration between the three groups of rats with 5/6, 2/3, and 1/2 nephrectomy. Our results appear to differ from the clinical trial, in which a correlation between the plasma copeptin concentration and CKD progression was revealed [24, 25]. It seems, however, that the increase of the copeptin plasma concentration in patients with chronic kidney disease was probably not related to the progression of CKD, but most likely related to other pathologies, including chronic heart failure and diabetes types 1 and 2 [25–27]. It has also been shown that a significant relationship exists between the concentrations of copeptin and NT-proBNP and B-type natriuretic peptide (BNP) in patients with chronic heart failure [28, 29]. In our study, NT-proBNP plasma concentrations were significantly higher only in the* N5/6* group compared with the* CN* group. There were no differences in the concentration of NT-proBNP and copeptin between the nephrectomized rats (*N1/2*,* N2/3*,* N5/6*). Several studies have shown a negative correlation between the plasma concentration of natriuretic peptide levels and GFR [30, 31]. In addition, it is suggested that increases in BNP and NT-proBNP in plasma may be associated with an increased risk of accelerated CKD to end-stage renal disease (ESRD) progression probably because heart failure develops [32]. It is known that copeptin concentration in plasma increases with increased osmolarity [33]. In the present study, the increase in plasma osmolarity in rats with 5/6 nephrectomy was not accompanied by increased plasma concentrations of copeptin. Copeptin concentration in plasma may be dependent not only on plasma osmolarity. Another factor which greatly determined copeptin concentration in plasma is the water content in the body. All rats used in the study had continuous access to drinking water, which could be the reason for the continuous release of copeptin from the hypothalamus, regardless of the concentration of the osmotically active compounds in the blood. These assumptions seem to confirm both experimental and clinical studies, which have found that the fluctuations of copeptin plasma concentration depend on the supply of water and the hydration of the body [34–36]. It is increasingly emphasized that, in the impaired conditions of renal function, the concentration of copeptin and vasopressin in plasma may not be equal, due to the different clearance of both peptides [37].

Based on the results we obtained, it can be assumed that chronic kidney disease significantly affects the changes in the expression of the vasopressinergic system in the kidney cortex in rats with nephrectomy. We observed that the expression of the mRNA V1a receptor in the kidney cortex was significantly lower in the* CN* group compared with the other groups. On the other hand, protein V1aR expression was significantly lower in the* N5/6* group in comparison with the other groups. It seems that the decrease in the V1a receptor protein level relative to the V1aR mRNA expression in the* N5/6* group could be caused by the increased activity of the vasopressinergic system. It has been proven that V1a receptors are internalized into the cell cytoplasm after being connected with vasopressin and then recycled, thanks to which they can again function as membrane receptors [38]. It is known that AVP causes a decrease in kidney function [37]. In addition, plasma levels of vasopressin increase with increasing progression of renal damage [39]. It seems that, in the present study due to the increase in the release of AVP into the circulation, there is an increased synthesis of V1a receptors in the kidney cortex, which is reflected in the increased expression of V1aR mRNA in the kidney cortex. The vasopressin released into the circulation can connect with the V1aR receptors causing their internalization, which has been demonstrated as a decrease in the level of the V1a receptor protein in the kidney cortex [40]. The role of the V1a receptors in the pathogenesis and progression of chronic kidney disease is not clear. Higashiyama et al. [41] showed that V1a receptors were involved in protecting the glomerular mesangial cells from apoptosis on the pathway dependent on intracellular protein kinase C (PKC). In contrast, Wind et al. [42] demonstrated the role of the V1a receptors in the process of contraction, proliferation, and glomerular mesangial cell hypertrophy, which secondarily decreases the glomerular filtration rate. On the other hand, administration of the selective V1a receptor antagonist (VRA) prevented the development of proteinuria and hypertension in the 5/6 nephrectomy rat model and proteinuria in nephrotic syndrome in the adriamycin rat model [43, 44]. It seems that the difference in these observations may be caused by a different V1aR activity in the various layers of the kidney cortex [45]. In our study, there was a significant decrease in eGFR in the* N5/6* group compared with the other groups, which was associated with a decrease in the level of the V1a receptor protein in the kidney cortex. Vasopressin acting directly on the V1a receptor located in the smooth muscle cells in the renal cortex may contribute to the development of renal dependent hypertension [46].

mRNA V2 receptor expression in the kidney cortex was significantly lower in the* CN* group compared with the other groups. The level of the V2R protein was significantly lower in the* N5/6* group compared with the* CN* group. The lower level of the V2 receptor protein could also be due to the smaller surface of the kidney cortex in the nephrectomized rats compared with the control animals. It seems that the potential cause of the differences in V2 receptor mRNA expression and V2R protein level in the kidney cortex could be, as with the V1a receptor, the process of internalizing the receptor into the cytoplasm due to the increased release of AVP into the circulation [47]. It is known that, under physiological conditions, vasopressin activates the V2 receptors in the collecting ducts and the thick ascending limb of the loop of Henle, increases the reabsorption of water, sodium, and urea, and facilitates the urine concentrating mechanism [46, 48]. Recent studies indicate the role of V2 receptors in the progression of chronic renal disease [39]. Our study showed significantly higher urea plasma levels in rats in the* N5/6* group in comparison with other groups of rats. These results may indicate an increased activity of V2 receptors in the kidney cortex in the* N5/6* group. The available literature data indicate that there are significant individual differences in the V2R dependent signaling pathways involved in the processes of reabsorption [48]. Probably, the water supply plays a significant role in this process. Bouby et al. [49] have demonstrated a lower percentage of glomeruli destroyed in rats with 5/6 nephrectomy, which had free access to drinking water and had water added to their feed, compared with rats, which had free access to drinking water and had no water added to their feed. Sugiura et al. [50] described a significant increase in the volume of urine and decrease in its osmolarity in rats with 5/6 nephrectomy, probably due to disturbances in the functioning of the V2 receptors. Also in our study, a significant decrease in urine osmolarity and a significant decrease in the concentration of sodium and urea were found in the* N5/6* group compared with other groups. Vasopressin regulates the body's water balance via specialized membrane proteins called aquaporins (AQP) [51]. Activation of the V2 receptor leads to the exocytosis of vesicles containing aquaporins 2 with apical membrane, the spinal and cortical collecting duct, facilitating the mechanism of urinary compaction and the transportation of water, sodium, and urea [52, 53]. In our study, the AQP2 protein level was significantly higher in the* CN* group as compared to the* N1/2* and* N2/3* groups. There were no significant differences in the AQP2 protein level between the* CN* group and the* N5/6* group. The low AQP2 protein level in nephrectomized rats was adequate for the low level of V2 receptor protein. Low level of V2R and AQP2 proteins was associated with a significant increase in urine volume in the* N5/6* compared with the other groups. Decreased levels of AQP2 protein in* N5/6* rats caused, most likely, a decrease in reverse reabsorption of water, which contributed to the increase in the urine volume, as well as the decrease in urine osmolarity, as evidenced by the low concentration of sodium and urea in urine. Our results are consistent with the observations of Kwon et al. [54], who suggested that an increase in urine volume and a decrease in osmolarity in rats with 5/6 nephrectomies that developed chronic renal failure (CRF) was due to decreased protein expression not only of AQP2, but also of AQP1 and AQP3. In our study, rats from all experimental groups had free access to water. Suzuki et al. [55] showed that a 24-hour water restriction in the CRF rats significantly reduced the volume of urine output and increased its osmolarity. In addition, these investigators have reported an increase in AQP2 mRNA associated with dehydration of rats, which may be one of the mechanisms of residual urinary compaction capacity in CRF. In our study in nephrectomized rats, a decrease in urine osmolarity was accompanied by an increase in plasma osmolarity. However, it seems that the increase in plasma osmolarity did not occur due to increased reabsorption of sodium, but due to the increased reabsorption of urea in the kidneys. It has been reported that vasopressin facilitates the transport of urea by affecting the increase in the activity of its transporters (UT), in particular the UT-A1 urea transporter [56]. It was documented that urea transporters (UT-A1, UT-A2, UT-A3) are located in the kidney medulla collecting duct [57, 58]. Unfortunately, due to the small amount of isolated kidney medulla, it was not possible to study the expression of urea transporters. Future studies on the expression of urea transporters should clarify the mechanism involved in the effect of vasopressin on the activity of urinary transporters in rats after 5/6 nephrectomy.

## 5. Conclusions

Chronic kidney disease caused significant changes in the expression of the vasopressinergic system. Copeptin does not appear to be a good CKD progression evaluation marker in the experimental model of nephrectomy without concomitant diseases. Further research is needed to explain the mechanisms of the impact of the vasopressinergic system on the kidney in chronic kidney disease.

## 6. Study Limitation

The study was performed on a small number of rats in individual experimental groups. This was due to the high invasiveness of the surgical procedures. Examination of the expression of mRNA and protein of the vasopressinergic receptors (V1a and V2) and AQP2 were carried out only in the kidney cortex. For technical reasons, it is difficult to get enough of the isolated RNA or protein from the kidney core for further determinations.

## Figures and Tables

**Table 1:** Biochemical parameters in the urine.

**Parameters**	***CN***	***N1/2***	***N2/3***	***N5/6***
	**(n=7)**	**(n=7)**	**(n=6)**	**(n=8)**
**urine volume (ml/24h)**	8,53 ± 0,54^*∗∗∗*^	14,21 ± 0,99^##^	15,90 ± 0,58^&^	25,10 ± 3,01
**osmolarity (mOsmol/kgH**_**2**_ **O) **	1786 ± 84,44^*∗∗*^	2004.0 ± 99.9^###@^	1589.8 ± 55.2^&^	1191.7 ± 108.3
**urea (mg/ml)**	7805.8 ± 558.4^*∗∗∗*$$₤₤^	5552.9 ± 420.7^###^	5235.0 ± 464.9^&&&^	1331.3 ± 162.8
**sodium (mmol/l)**	92.1 ± 6.3^*∗∗∗*^	86.9 ±5.0^###^	69.6 ± 7.6	53.0 ± 8.5
**creatinine (mg/dl)**	110.8 ± 8.1^*∗∗∗*^	95.7 ± 5.7^###^	106.0 ± 12.0^&&&^	29.5 ± 6.0
**protein (mg/dl)**	148.7 ± 8.0	345.1 ± 24.9	1780.0 ± 577.4	705.9 ± 101.5^*∗∗∗*#^
**protein/creatinine**	1.4 ± 0.1	3.7 ± 0.4	19.6 ±7.7^$^	65.3 ± 33.5^*∗∗∗*#^

Biochemical parameters of urine. *CN*: control group; *N1/2*: 1/2 nephrectomy group; *N2/3*:2/3 nephrectomy group; *N5/6*: 5/6 nephrectomy group. Means ± SE are shown. *∗*Significant difference between *N5/6 *and *CN* groups; ^#^significant difference between *N5/6 *and *N1/2* groups; ^&^significant difference between *N5/6 *and *N2/3* groups; ^@^significant difference between *N2/3 *and *N1/2* groups; ^$^significant difference between *N2/3 *and *CN* groups; ^₤^significant difference between *N1/2 *and *CN* groups. *∗∗*P<0.01; *∗∗∗*P<0.001; ^#^P<0.05; ^###^P<0.001; ^&^P<0.05; ^&&&^P<0.001; ^@^P<0.05; ^$^P<0.05; ^$$^P<0.01; ^₤₤^P<0.01.

**Table 2:** Biochemical parameters in the plasma.

**Parameters**	***CN***	***N1/2***	***N2/3***	***N5/6***
	**(n=7)**	**(n=7)**	**(n=6)**	**(n=8)**
**osmolarity (mOsmol/kgH**_**2**_ **O) **	300 ± 2.9	338 ± 13.3^₤^	351 ± 7.9^$$^	335 ± 7.5^*∗*^
**urea (mg/ml)**	32.3 ± 1.3	44.6 ± 2.3	67.1 ± 5.3	75.3 ± 3.6^*∗∗∗*##^
**sodium (mmol/l)**	140.6 ± 0.8^₤^	130.0 ± 1.0	138.5 ± 2.7	123.7 ± 16.8
**creatinine (mg/dl)**	0.3 ± 0.0	0.6 ± 0.0	0.7 ± 0.1	9.2 ± 8.4^*∗∗∗*##&&^
**eGFR (ml/min)**	2.3 ± 0.2^*∗∗∗*###&&$₤^	1.6 ± 0.1^###^	1.5 ± 0.2^&&^	0.6 ± 0.1
**cystatin C (pg/ml)**	2992.4 ± 131.2	3477.9 ± 314.0	4360.2 ± 344.6^$^	5708.9 ± 345.4^*∗∗∗*##&&^
**NT-proBNP (pg/ml)**	131.2 ±17.5	288.5 ± 143.7	259.3 ± 97.1	378.9 ± 71.4^*∗*^

Biochemical parameters evaluated in the plasma. *CN*: control group; *N1/2*: 1/2 nephrectomy group; *N2/3*:2/3 nephrectomy group; *N5/6*: 5/6 nephrectomy group; eGFR: estimated glomerular filtration rate; NT-proBNP: N-terminal brain natriuretic peptide. Means ± SE are shown. *∗*Significant difference between *N5/6 *and *CN* groups; ^#^significant difference between *N5/6 *and *N1/2* groups; ^&^significant difference between *N5/6 *and *N2/3* groups; ^$^significant difference between *N2/3 *and *CN* groups; ^₤^significant difference between *N1/2 *and *CN* groups. *∗*P<0.05; *∗∗∗*P<0.001; ^##^P<0.01; ^###^P<0.001; ^&&^P<0.01; ^$^P<0.05; ^$$^P<0.01; ^₤^P<0.01.

## Data Availability

All data supporting the results reported in the article can be found in Department of Experimental and Clinical Physiology, Laboratory of Center for Preclinical Research, Medical University of Warsaw and can be seen after asking the corresponding author.
